# Hepatocyte GPCR signaling regulates IRF3 to control hepatic stellate cell transdifferentiation

**DOI:** 10.1186/s12964-023-01416-6

**Published:** 2024-01-17

**Authors:** Jae-Hyun Yu, Myeung Gi Choi, Na Young Lee, Ari Kwon, Euijin Lee, Ja Hyun Koo

**Affiliations:** College of Pharmacy and Research Institute of Pharmaceutical Sciences, Seoul National University, Seoul, 08826 Republic of Korea

**Keywords:** GPCR, IRF3, IL-33, Gαs, PKA, Hepatic stellate cell

## Abstract

**Background:**

Interferon Regulatory Factor 3 (IRF3) is a transcription factor that plays a crucial role in the innate immune response by recognizing and responding to foreign antigens. Recently, its roles in sterile conditions are being studied, as in metabolic and fibrotic diseases. However, the search on the upstream regulator for efficient pharmacological targeting is yet to be fully explored. Here, we show that G protein-coupled receptors (GPCRs) can regulate IRF3 phosphorylation through of GPCR-Gα protein interaction.

**Results:**

IRF3 and target genes were strongly associated with fibrosis markers in liver fibrosis patients and models. Conditioned media from MIHA hepatocytes overexpressing IRF3 induced fibrogenic activation of LX-2 hepatic stellate cells (HSCs). In an overexpression library screening using active mutant Gα subunits and Phos-tag immunoblotting, Gαs was found out to strongly phosphorylate IRF3. Stimulation of Gαs by glucagon or epinephrine or by Gαs-specific designed GPCR phosphorylated IRF3. Protein kinase A (PKA) signaling was primarily responsible for IRF3 phosphorylation and Interleukin 33 (IL-33) expression downstream of Gαs. PKA phosphorylated IRF3 on a previously unrecognized residue and did not require reported upstream kinases such as TANK-binding kinase 1 (TBK1). Activation of Gαs signaling by glucagon induced IL-33 production in hepatocytes. Conditioned media from the hepatocytes activated HSCs, as indicated by α-SMA and COL1A1 expression, and this was reversed by pre-treatment of the media with IL-33 neutralizing antibody.

**Conclusions:**

Gαs-coupled GPCR signaling increases IRF3 phosphorylation through cAMP-mediated activation of PKA. This leads to an increase of IL-33 expression, which further contributes to HSC activation. Our findings that hepatocyte GPCR signaling regulates IRF3 to control hepatic stellate cell transdifferentiation provides an insight for understanding the complex intercellular communication during liver fibrosis progression and suggests therapeutic opportunities for the disease.

Video Abstract

**Supplementary Information:**

The online version contains supplementary material available at 10.1186/s12964-023-01416-6.

## Introduction

IRF3 is a transcription factor which is originally identified to be responsible for relaying signals from upstream factors recognizing foreign antigens by initiating cellular defense mechanisms through interferon transcription [1]. Recent studies suggest that IRF3 may also play a role in other biological processes, such as the regulation of metabolism, cell proliferation, and differentiation. For instance, IRF3 regulates cell cycle genes and inhibits cell growth [2]. Moreover, IRF3 is shown to be increased during adipocyte differentiation process [3]. Ablation of IRF3 has been shown to increase thermogenesis [4]. Nonetheless, the exact mechanisms by which IRF3 regulates these processes are still not fully understood, and it is needed to elucidate the mechanisms underlying on the regulations and functions of IRF3.

In the liver, IRF3 and its target genes exert a wide range of biological function, including innate immune response, gluconeogenesis and apoptosis [5–10]. Abnormal increase in IRF3 activity in hepatocytes has been linked with diseases, particularly in liver fibrosis. For instance, livers from fibrosis patients show increased IRF3 expression and nuclear translocation in hepatocytes when compared to those from healthy volunteers [9, 11]. An experimental model of liver fibrosis using chronic administration of carbon tetrachloride also results in IRF3 activation [12]. On the other hand, IRF3 knockout mice are resistant to develop liver fibrosis in the same model [13]. However, how hepatocyte IRF3 regulates liver fibrosis or trandifferentiation of HSCs, the cells primarily responsible for fibrogenesis in the liver [14, 15], is poorly understood. One potential link would be IL-33, a target gene of IRF3 [16]. IL-33 is known to activate HSCs, as indicated by increases in *Acta2*, *Timp1* and *Col1a1* [17, 18]. Moreover IL-33 levels are increased in fibrotic livers of both humans and mice [17]. Mechanistically, IL-33 binds to the ST2 receptor then activates signaling pathways such as NF-κB and mitogen-activated protein kinases, which are important for HSC transdifferentiation [19]. Indeed, ST2 deficiency in HSCs abrogates IL-33-induced fibrosis progression [18]. Therefore, IRF3 may be an attractive therapeutic target for liver fibrosis which interferes with pathological intercellular communication in the liver.

IRF3 activity is mostly determined by its phosphorylation status. For instance, it is phosphorylated by TBK1 in response to viral infections and inflammatory cytokines. TBK1 phosphorylate multiple specific serine and threonine residues in the C-terminal domain of IRF3. Phosphorylation leads to its homodimerization which in turn results nuclear translocation and target gene transactivation. Recent studies have identified several other kinases that can phosphorylate IRF3, including IκB kinase ε (IKKε), c-Jun-N-terminal kinase (JNK) and mammalian sterile 20-like kinase 1 (MST1) [20–22]. Despite this information, research on the upstream factors that regulate IRF3 is still warrants further discovery.

GPCRs are membrane proteins that play a crucial role in receiving external signals and transmitting them downstream via Gα proteins. Humans have about 800 GPCRs, but only 21 Gα proteins [23]. Gα proteins are categorized as Gαs, Gαi, Gαq, and Gα12 and are activated when GDP is converted to GTP [24]. GPCRs offer great potential for drug discovery and development. To date, GPCRs serve as drug targets for majority of therapeutic drugs since they regulate a wide variety of physiological processes and provide accessible drug sites on the cell surface, without requiring the drug to be diffused inside the target cell. Here, we demonstrate that GPCR signaling regulates IRF3 phosphorylation in hepatocytes and that the regulation of IRF3 by Gαs-PKA signaling induces IL-33 expression to promote HSC transdifferentiation.

## Materials and methods

### Animal experiment

Seven-week-old male C57BL/6J mice were fed with choline-deficient, L-amino acid-defined, high-fat diet (CDAHFD) (A06071302, Research Diets) for 0, 2, 4, or 12 weeks and then sacrificed (*n* = 6 each). For hyperphagic obese model, 12-week-old *db*/*db* mice were fasted for 8 h before sacrifice. Mice were bred and maintained under specific pathogen-free conditions with a 12-hour dark/12-hour light cycle and controlled temperature and humidity. The study was approved by the Institutional Animal Care and Use Committee of Seoul National University and the Catholic University of Korea, respectively.

### Antibodies and reagents

Anti-Flag (#F1804) and anti-Vinculin (#V9131) antibodies were from Sigma. Antibodies against IRF3 (#sc-33641), p-CREB1 (#sc-81,486), IRF7 (#sc-74471), Vimentin (#sc-32322) were obtained from Santa Cruz. α-SMA antibody were from Abcam (#ab7817). IL-33 (AF3626) antibody was from R&D systems. LATS1 (#3477), HA-tag (#2367) and Lamin A/C (#2032) antibodies were from Cell Signaling. Epinephrine (#E4250) was from Sigma. Phos-tag acrylamide (#AAL-107) was from Wako Chemicals. Glucagon (#24204), Forskolin (#11018) and BX795 (#14932) were from Cayman. H89 (#S1582) was from Selleckchem.

### Cell culture and DNA transfection

Mouse primary hepatocytes were isolated by in situ collagenase perfusion method. The cells were maintained in Willam’s medium E (Welgene) and used for experiments after 16 h. HepG2, MIHA, LX-2, HEK293, HEK293T cell lines were maintained in DMEM (Welgene). For DNA transfection, cells were transfected with plasmids using PolyJet in vitro transfection reagent (Signagen) for 6 h, recovered with fresh media for 18 h, then used for experiments.

### Cloning

The genes coding for IRF3, TBK1, PKA and ADRB2 was amplified from HEK293 cDNA as a template. GCGR was amplified from GCGR-Tango (Addgene, #66,291). cDNA fragments were cloned into pRK7 (Addgene, #10,883) or pCDH-EF1-FHC (Addgene, #64,874) by Gibson assembly reaction using NEBuilder HiFi DNA assembly master mix (New England Biolabs). After purification, all plasmids were verified by Sanger sequencing.

### Quantitative real-time PCR (qRT-PCR)

Total RNA was isolated using RNeasy Mini kit (Qiagen) for cell lines or TRIzol reagent (Invitrogen) for mouse livers. Reverse transcription was done using AccuPower RT premix (Bioneer). qRT-PCR was performed using AccuPower 2X GreenStar qPCR Master Mix (Bioneer). After PCR amplification, melt curve of each amplicon was obtained to verify its accuracy. All mRNA levels were normalized to expression of *GAPDH* level. Primer sequences used for PCR are available in Supplemental Table 1.

### Immunoblot analysis

Polyacrylamide gels containing Phos-tag acrylamide were prepared according to the manufacturer’s instructions. Cell or tissue lysates were quantified for protein content and separated by SDS-PAGE. The proteins were then transferred to nitrocellulose membranes (GE Healthcare). Proteins of interest were probed with primary antibodies and horse-radish peroxidase-linked secondary antibodies for chemiluminescence detection.

### Conditioned media

Mouse primary hepatocytes maintained in William’s media E without serum for 24 h after isolation were treated with glucagon (10 nM) for 24 h. The conditioned media were collected filtered through 0.22 μm polyethersulfone filter. Proteins were purified and concentrated using 10 kDa-cutoff Amicon Ultra centrifugal filter [25].

### Statistical analysis

All statistical analysis was performed using SigmaPlot. Criteria for statistical significance sere considered to be significant when *p < 0.05 and **p < 0.01.

## Results

### IRF3 expression is associated with liver fibrosis progression

**Fig. 1:**
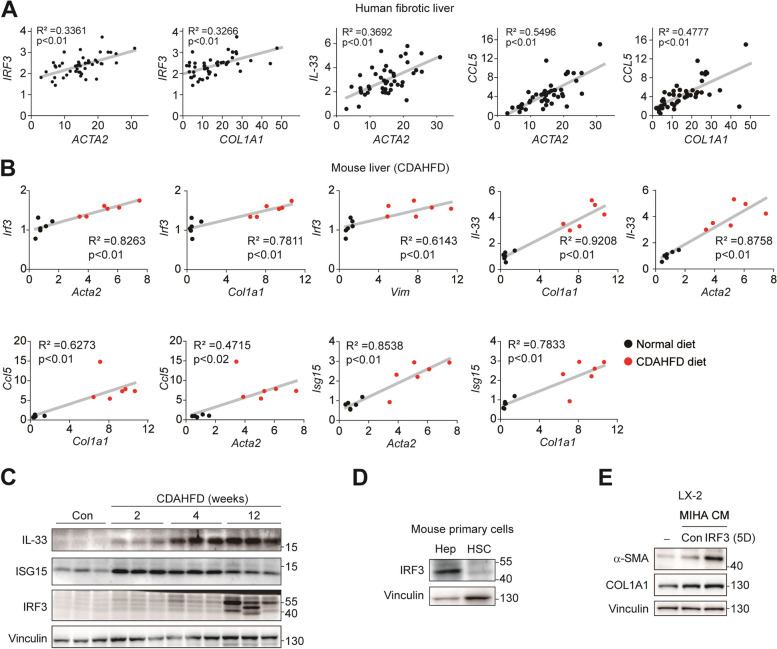
IRF3 expression is associated with liver fibrosis progression. **A** Correlation between *IRF3* and its target genes *(IL-33* and *CCL5*) and fibrosis markers (*ACTA2* and *COL1A1*) mRNA levels in fibrotic human livers or heathy livers. **B** Correlation comparisons between *IRF3* and its target genes (*Il-33*, *Ccl5*, and *Isg15*) and fibrosis markers (*Acta2*, *Col1a1*, and *Vim*) mRNA levels in chow diet and CDAHFD diet. **C** Western blot analysis of IL-33 and ISG15 and IRF3 in wild-type (WT) mice (*n* = 3 each) treated with chow or CDAHFD. **D** Western blot analysis of IRF3 in mouse primary hepatocytes and primary hepatic stellate cells. **E** Immortalized human hepatocytes MIHA were transfected with IRF3 (5D) and secreted proteins were collected, proteins were concentrated and treated to LX-2 for 24 h

To determine the role of hepatic IRF3 in liver fibrosis, we first analyzed the transcriptomic profiles of fibrosis patients (GSE25097). The expression of IRF3 and IRF3 target genes, namely *IL-33* and *CCL5*, positively correlated with the levels of pro-fibrotic markers *α-SMA* (or *ACTA2*) and *Col1A1* (Fig. 1A). Also, a mouse liver fibrosis cohort induced by CDAHFD feeding (GSE200409) showed similar results. Consistent with the observation from human fibrosis, IRF3 and IRF3 downstream genes (*Il-33*, *Isg15* and *Ccl5)* expression had significant positive correlations with pro-fibrotic markers in mice (Fig. 1B). Next, we sought to confirm the results from transcriptomic analysis using a temporal CDAHFD diet model. Since CDAHFD feeding results in a stepwise development of non-alcoholic fatty liver disease (i.e. steatosis, hepatitis, and fibrosis) over time, mice were sacrificed at different time points ranging from 2 weeks to 12 weeks to determine the temporal pattern of protein expression during fibrosis development and progression. As a result, CDAHFD diet increased the protein expression of IRF3 and the expression of IRF3-activated downstream proteins after 4 weeks, which is the point having hepatitis and some fibrosis (Fig. 1C). Of note, IRF3 protein was exclusively expressed in mouse primary hepatocytes when compared with mouse primary hepatic stellate cells (Fig. 1D), implying that hepatocytes are the primary sites for IRF3 activation in the liver. Indeed, conditioned media from MIHA (immortalized hepatocytes) cells expressing active mutant IRF3 promoted myofibroblastic transdifferentiation of LX-2 (HSCs) cells as indicated by increased expression of α-SMA and COL1A1 as the marker proteins (Fig. 1E). Our results show that IRF3 is involved in liver fibrosis and activates hepatic stellate cells through intercellular signaling.

### Gαs-coupled GPCR signaling phosphorylates IRF3

**Fig. 2:**
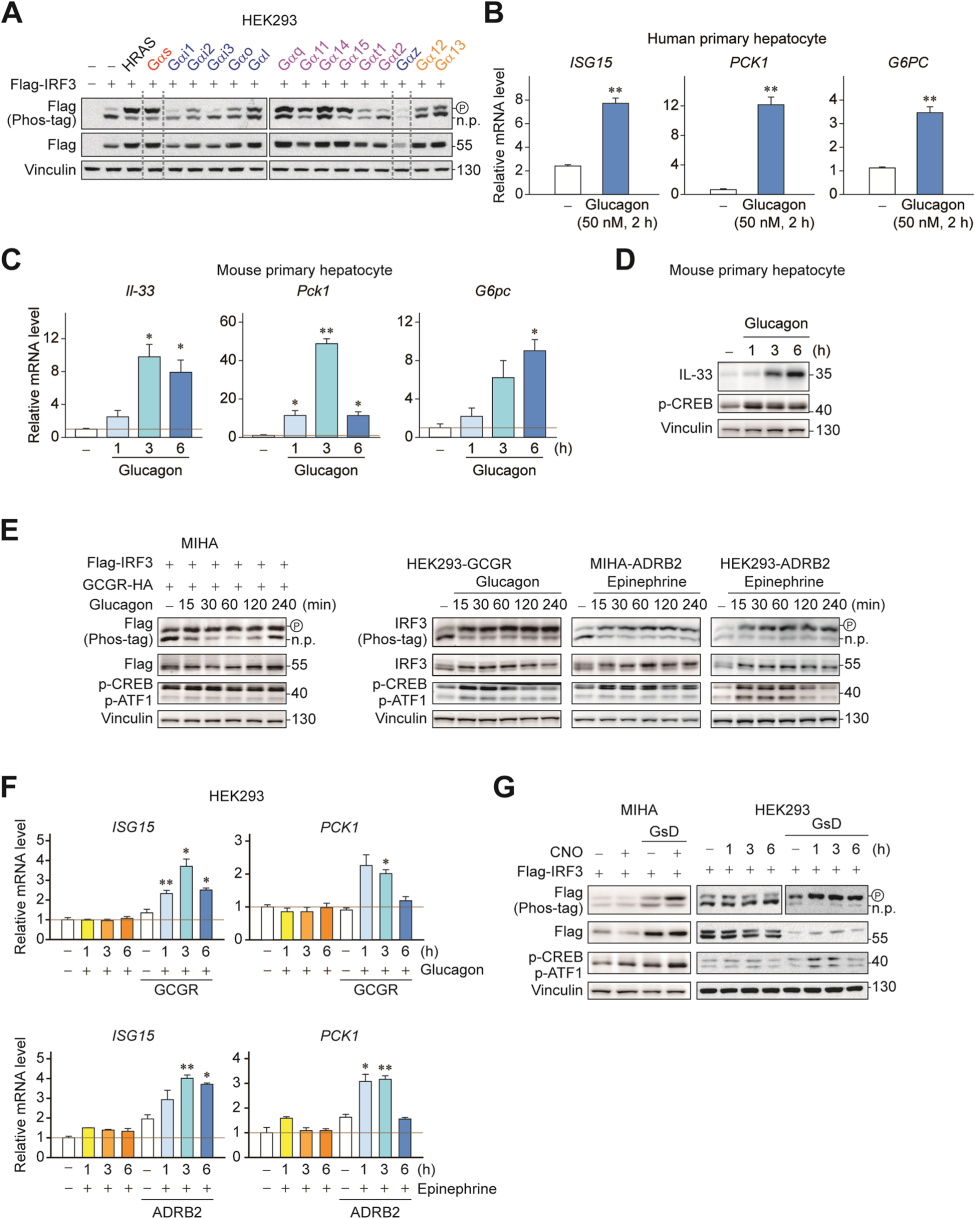
Gαs-coupled GPCR signaling phosphorylates IRF3. **A** HEK293 cells were co-transfected with 200 ng of Flag-IRF3 and 200 ng of Gα subunits, respectively. 24 h post transfection, the migration shift of IRF3 was determined by Phos-tag gel electrophoresis. Red, Gαs; Blue, Gαi/o; Violet, Gαq/11; and Yellow, Gα12/13 subfamilies, respectively. **B** mRNA level of *ISG15*, *PCK1* and *G6PC* in human primary hepatocytes with or without glucagon 2 h treatment. **C** Mouse primary hepatocytes were treated with 10 nM of glucagon for 0, 1, 3, 6 h. RT-qPCR was used to detect expression of *Il-33*, *Pck1* and *G6pc*. **D** Mouse primary hepatocytes were treated with 10 nM of glucagon for 0, 1, 3, 6 h. **E** MIHA cells were co-transfected with 200 ng of Flag-IRF3 and 50 ng of GCGR-HA. 24 h post transfection, the cells were treated with 10 nM of glucagon for indicated times. HEK293-GCGR, HEK293-ADBR2 and MIHA-ADRB2 stable cells were treated with 10 µM of epinephrine or 10 nM of glucagon for indicated times. **F** HEK293 cells were transfected with 800 ng of HA-ADRB2 or 200 ng of GCGR-HA or empty plasmid, respectively. 24 h post transfection, the cells were treated with 50 µM epinephrine or 100 nM of glucagon for indicated times. RT-qPCR was used to detect expression of *ISG15* and *PCK1*. **G** MIHA and HEK293 cells were co-transfected with 200 ng of Flag-IRF3 and 200 ng of GsD or empty plasmid, respectively. 24 h post transfection, the cells were treated with 10 µM or 20 µM of CNO for indicated time, respectively. Statistical data were expressed as mean ± SEM; *p < 0.05 and **p < 0.01

GPCRs represent one of the largest gene families in our genome. Although there are over a thousand in number, they are coupled to only 4 types of different Gα proteins [23, 24]. To investigate the potential role of GPCR signaling in regulation, we screened different Gα proteins using GTPase-deficient, constitutively active mutants. Notably, phosphorylation of IRF3 was distinctively increased in cells transfected with Gαs, as indicated by Phos-tag immunoblotting (Fig. 2A). HRAS was used as a positive control, since it showed a robust IRF3 phosphorylation after overexpression in our previous study [26]. Analysis of transcriptomic data (GSE68144) revealed that Gαs activation by glucagon increases both IRF3 target (*ISG15*) and gluconeogenic genes (*PCK1* and *G6PC*) in human primary hepatocytes (Fig. 2B). Our results also have confirmed that glucagon treatment time-dependently induced *Il-33* along with *Pck1* and *G6pc* in mouse primary hepatocytes (Fig. 2C). Consistently, induction of glucagon also increased IL-33 protein level in mouse primary hepatocytes (Fig. 2D). We also tested whether hyperactivation of Gα-specific GPCR signaling can produce similar results. Receptors for glucagon or epinephrine (GCGR, glucagon receptor; or ADRB2, β2-adrenergic receptor, respectively), the representative Gαs-coupled receptors which promote gluconeogenesis in hepatocytes, were stably integrated into MIHA and HEK293 cells since cell lines do not respond to glucagon and epinephrine. Glucagon or epinephrine treatment with respective induction of GCGR or ADRB2 were able to induce IRF3 phosphorylation (Fig. 2E). Consistent with the results, an increase of IRF3 transcriptional activity was detected through upregulation of its target transcript *ISG15*, along with *PCK1* (Fig. 2F). GPCRs often engage with different types of Gα proteins per same receptor and produce mixed downstream signaling events. To specifically address the role of Gαs signaling downstream of ligand-receptor interaction, we used a rationally designed GPCR that only couples with Gαs (GsD) in response to a synthetic ligand, clozapine N-oxide [27]. As expected, clozapine N-oxide stimulated IRF3 phosphorylation in GsD-expressing cells (Fig. 2G). These data collectively showed that the signal generated by Gαs-coupled GPCRs increases IRF3 phosphorylation.

### Gαs signals through cAMP-mediated IRF3 phosphorylation and activation

**Fig. 3:**
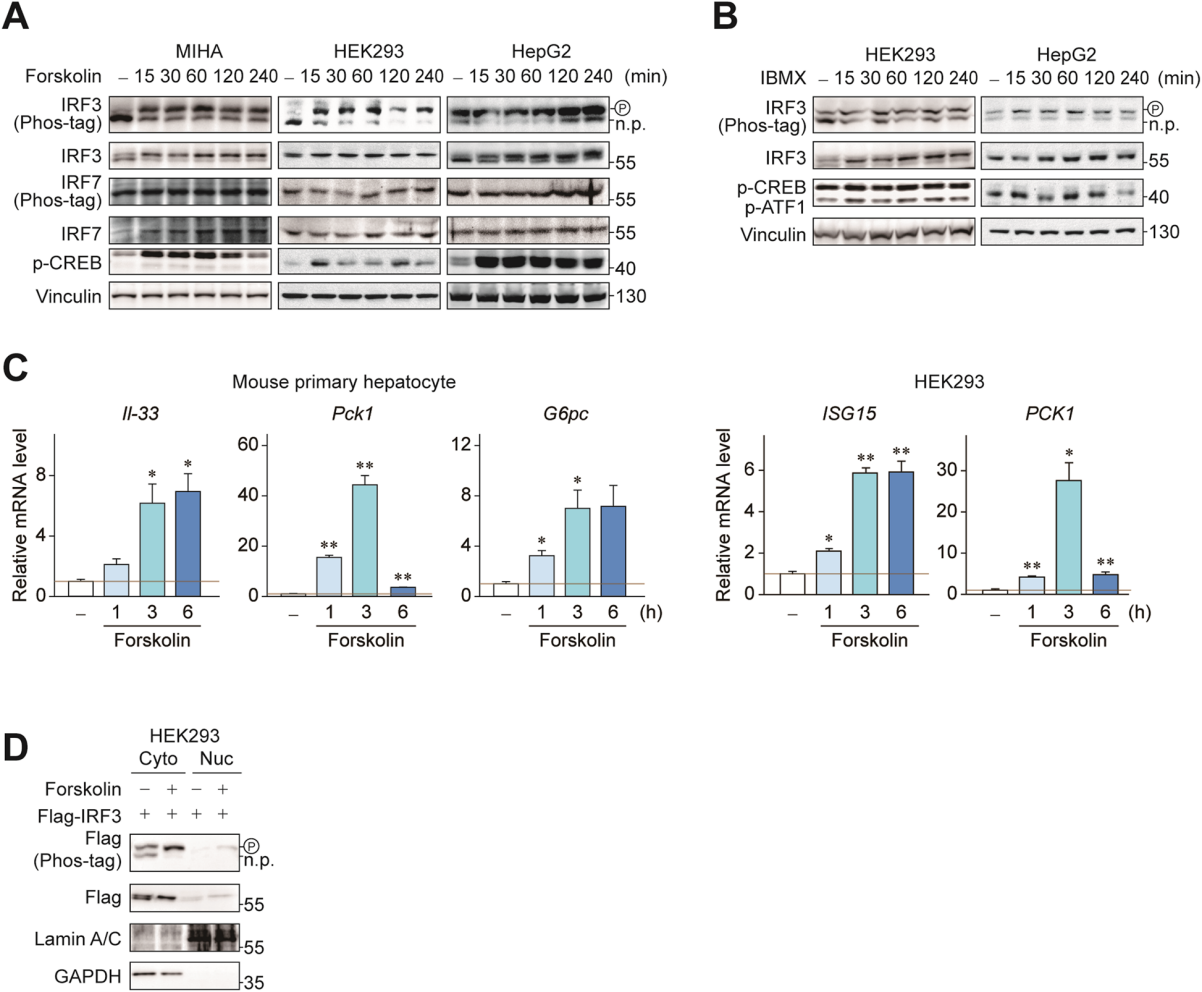
Gαs signals through cAMP-mediated IRF3 phosphorylation and activation. **A** MIHA, HEK293 cells and HepG2 cells were treated with 30 µM forskolin for indicated times. **B** HEK293 and HepG2 cells were treated with 500 µM of IBMX for indicated times. Phosphorylation of IRF3 was determined by Phos-tag immunoblottings. **C** Mouse primary hepatocytes and HEK293 cells were treated with 30 µM forskolin for indicated times. RT-qPCR was used to detect expression of *Il-33*, *Pck1* and *G6pc* in mouse primary hepatocytes and *ISG15* and *PCK1* in HEK293. **D** HEK293 cells were treated with 30 µM forskolin for 3 h. Nuclear/cytosolic fractionation were assessed by western blotting against specific markers Lamin A/C and GAPDH. Statistical data were expressed as mean ± SEM; *p < 0.05 and **p < 0.01

Activated Gαs increase intracellular second messenger cAMP through adenylyl cyclase activation. We investigated whether cAMP signaling is responsible for IRF3 phosphorylation. Stimulation of adenylyl cyclase with forskolin treatment increased phosphorylation of IRF3 in a time-dependent manner (Fig. 3A). On the other hand, phosphorylation of IRF7 which share high amino acid sequence homology with IRF3 was not altered. Treatment of cells with the phosphodiesterase inhibitor IBMX also increased IRF3 phosphorylation, which further confirmed the role of cAMP (Fig. 3B). Similar results are obtained with an increase in IRF3 activity, as indicated by induction of *Il-33* and *ISG15* mRNA expression in mouse primary hepatocytes and HEK293 (Fig. 3C). IRF3 is known to shuttle from cytoplasm to the nucleus upon phosphorylation on multiple amino acid residues by TBK1. However, immunoblottings using subcellular fractions showed that forskolin treatment induced phosphorylation of both cytoplasmic and nuclear IRF3 (Fig. 3D), implying that the phosphorylation observed through phos-tag mobility shift is regardless of IRF3 nuclear localization. These data showed that increased cAMP mediated IRF3 phosphorylation and activation, in a previously uncharacterized manner.

### PKA increases IRF3 phosphorylation and nuclear translocation

**Fig. 4:**
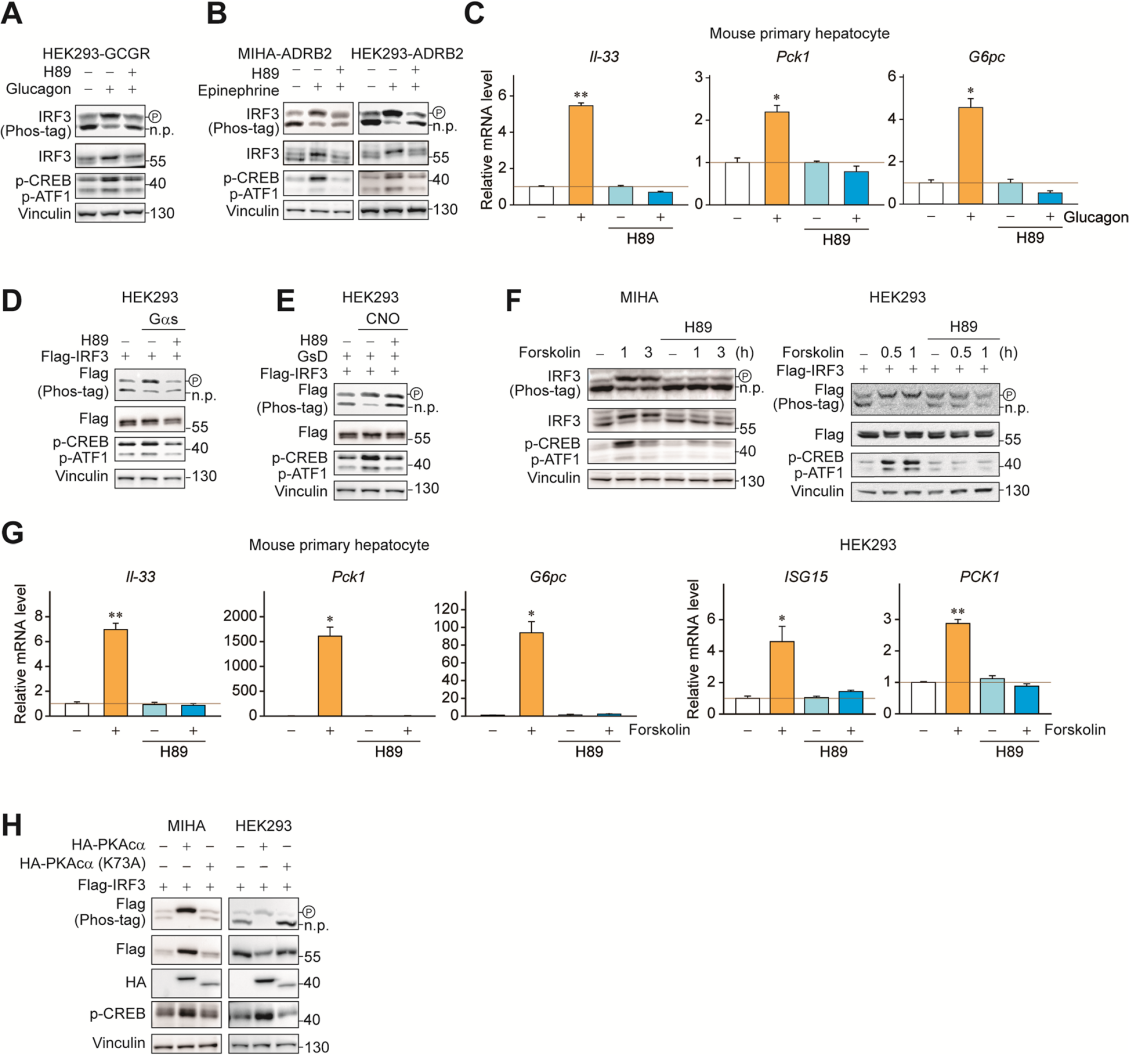
PKA signaling is responsible for IRF3 phosphorylation and activation. **A** HEK293-GCGR stable cells were treated for 1 h in the presence or absence of 10 nM Glucagon in the presence or absence of 10 µM H89 for 1 h. The migration shift of IRF3 was determined by Phos-tag gel electrophoresis. **B** MIHA-ADRB2 and HEK293-ADRB2 cells were treated for 1 h in the presence or absence of 10 µM epinephrine in the presence or absence of 10 µM H89 for 1 h. **C** Mouse primary hepatocytes were treated with or without glucagon (10 nM, 3 h) in the presence or absence of H89 (10 µM) for 1 h. RT-qPCR was used to detect expression of *Il-33*, *Pck1* and *G6pc*. **D** HEK293 cells were co-transfected with 100 ng of Flag-IRF3 and 200 ng of HA-Gαs, respectively. 24 h post transfection, cells were treated with or without H89 (10 µM, 1 h). **E** HEK293 cells were co-transfected with 200 ng of Flag-IRF3 and 200 ng of GsD, respectively. 24 h post transfection, cells were treated with or without clozapine-N-oxide (CNO; 1 µM, 1 h) in the presence or absence of H89 (10 µM) for 1 h. **F** MIHA cells were treated with or without forskolin (30 µM) in the presence or absence of H89 (10 µM) for 1 h. HEK293 cells were treated with or without forskolin (30 µM) in the presence or absence of H89 (10 µM) for 1 h. **G** Mouse primary hepatocytes were treated with or without forskolin (30 µM, 3 h) in the presence or absence of H89 (10 µM) for 1 h. RT-qPCR was used to detect expression of *Il-33*, *Pck1* and *G6pc*. HEK293 cells were treated with or without forskolin (30 µM, 1 h) in the presence or absence of H89 (10 µM) for 1 h. RT-qPCR was used to detect expression of *ISG15* and *PCK1*. **H** Phos-tag immunoblotting for Flag-tagged IRF3. MIHA and HEK293 cells were co-transfected with 200 ng of Flag-IRF3 and 400 ng of HA-PKAcα or HA-PKAcα (K73A) or empty, respectively. Statistical data were expressed as mean ± SEM; *p < 0.05 and **p < 0.01

Increased cAMP exerts many physiological processes which is mainly regulated by either PKA or exchange protein activated by cAMP (EPAC). Therefore, we have tested whether PKA inhibition can prevent IRF3 phosphorylation induced by conditions that activates Gαs and downstream signaling. Indeed, treatment with H89, a chemical inhibitor of PKA, blocked IRF3 phosphorylation induced by glucagon or epinephrine (Fig. 4A, B). Consistently, glucagon-induced induction of *Il-33*, *Pck1*, and *G6pc* was abolished by H89 in mouse primary hepatocytes as well (Fig. 4C). In addition, H89 also prevented IRF3 phosphorylation induced by CNO-mediated Gαs activation in GsD-expressing cells (Fig. 4D, E). H89 also blocked forskolin-induced IRF3 phosphorylation (Fig. 4F). Consistent with the results, induction of *Il-33* and *ISG15* by forskolin was abolished as well as gluconeogenic genes in mouse primary hepatocyte and HEK293 (Fig. 4G). To further confirm the role of PKA in IRF3 phosphorylation and rule out possible chemical off-target effects, the catalytic subunit of PKA (PKAca) and IRF3 were co-expressed. As expected, PKAca introduction increased IRF3 phosphorylation. On the contrary, kinase-dead mutant of PKAca (K73A) failed to phosphorylate IRF3 (Fig. 4H). These data suggest that Gαs-cAMP-PKA signaling controls post-translational modification and transcriptional activity of IRF3.

### PKA induces tyrosine phosphorylation of IRF3

**Fig. 5:**
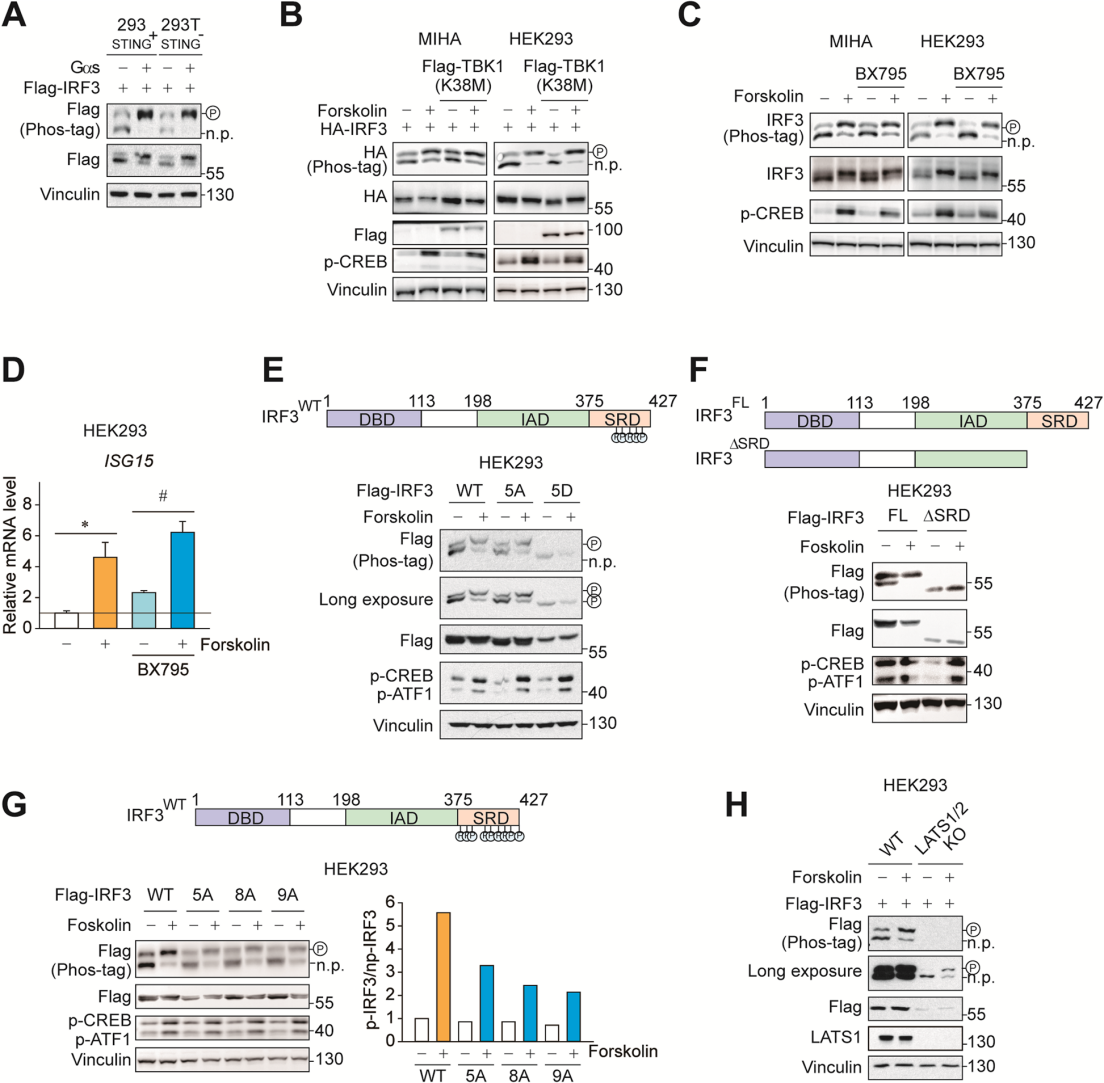
PKA induces tyrosine phosphorylation of IRF3. **A** HEK293 and HEK293T cells were co-transfected with 200 ng of Flag-IRF3 and 200 ng of HA-Gαs, respectively. 24 h post transfection, the migration shift of IRF3 was determined by Phos-tag gel electrophoresis. **B** MIHA and HEK293 cells were co-transfected with 200 ng of HA-IRF3 and 400 ng of Flag-TBK1 (K38M), respectively. 24 h post transfection, cells were treated with or without forskolin (30 µM, 1 h). **C** MIHA and HEK293 cells were treated with or without forskolin (30 µM, 1 h) in the presence or absence of BX795 (1 µM) for 1 h. The migration shift of IRF3 was determined by Phos-tag gel electrophoresis. **D** HEK293 cells were treated with or without forskolin (30 µM, 1 h) in the presence or absence of BX795 (10 µM) for 3 h. RT-qPCR was used to detect expression of *ISG15*. **E** HEK293 cells were transfected with 400 ng of Flag-IRF3 (WT) or Flag-IRF3 (5 A) or Flag-IRF3 (5D), respectively. 24 h post transfection, cells were treated with or without forskolin (30 µM, 1 h). **F** HEK293 cells were transfected with 400 ng of Flag-IRF3 (WT) or Flag-IRF3 (1-375), respectively. 24 h post transfection, cells were treated with or without forskolin (30 µM, 1 h). **G** HEK293 cells were transfected with 400 ng of Flag-IRF3 (WT) or Flag-IRF3 (5 A) or Flag-IRF3 (8 A) or Flag-IRF3 (9 A), respectively. 24 h post transfection, cells were treated with or without forskolin (30 µM, 1 h). **H** LATS1/2 knockout or wild-type HEK293 cells were transfected with 200 ng of Flag-IRF3 (WT), respectively. 24 h post transfection, cells were treated with or without forskolin (30 µM, 1 h). Statistical data were expressed as mean ± SEM; *p < 0.05 and **p < 0.01

In immune cells, IRF3 is phosphorylated by TBK1 downstream of cGAS-STING pathway in response to the recognition of nucleic acids in the cytoplasm [28]. We thus interrogated whether STING and TBK1 is responsible for PKA-mediated IRF3 regulation. HEK293T cells are genetically STING-deficient, while their parent cells, HEK293, do express STING [29]. Phosphorylation of IRF3 by constitutively active mutant Gαs was comparable in both cell lines, showing that STING is not required (Fig. 5A). Moreover, overexpression of kinase-dead mutant TBK1 (K38M; [30]) failed to prevent forskolin-mediated IRF3 phosphorylation (Fig. 5B). Consistently, TBK1 inhibitor BX795 also could not fully block IRF3 phosphorylation by PKA activation (Fig. 5C). The findings were further confirmed by mRNA induction of ISG15 by forskolin which was not affected by BX795 treatment (Fig. 5D). TBK1 phosphorylates 5 amino acid residues of IRF3 which are located in serine-rich domain near C-terminus [31]. Notably, the “non-phosphorylatable” mutant having all TBK1 substrate residues substituted to alanine still showed a mobility shift in Phos-tag gel by forskolin treatment (Fig. 5E). Moreover, phosphor-mimic mutant with aspartate substitution also showed some degree of mobility shift. These results show that PKA signaling induces phosphorylation on a previously unrecognized site on IRF3, regardless of TBK1. Next, we attempted to identify the sites of PKA-mediated IRF3 phosphorylation. The serine-rich domain (SRD) contains a cluster of reported phosphorylation residues that enhance transcriptional activity [32]. Truncation of SRD region abolished IRF3 phosphorylation after forskolin treatment, confirming SRD as the site of phosphorylation (Fig. 5F). We have further tested every amino acid residue in SRD with a potential for phosphorylation via site-directed mutagenesis. Substitution of serine or threonine sites (S385, S386, T390, S396, S398, S402, T404, S405 and S427) to alanine reduced Phos-tag mobility shift (Fig. 5G). Nevertheless, mutating all nine Ser/Thr residues together was insufficient to completely block phosphorylation of IRF3, implying the existence of additional phosphorylation sites comprised of other amino acids such as Tyr and His. Recently, it has been reported that activation of the LATS1/2 kinase by forskolin may increase phosphorylation of IRF3 [33]. However, in our assay system LATS1/2 deletion could not block forskolin-induced IRF3 phosphorylation (Fig. 5H). Given that PKA is only able to phosphorylate Ser and Thr residues directly, these data suggest that there is a kinase that relays PKA signaling for IRF3 phosphorylation.

### Gαs signaling in hepatocytes promotes HSC transdifferentiation through IL-33

**Fig. 6:**
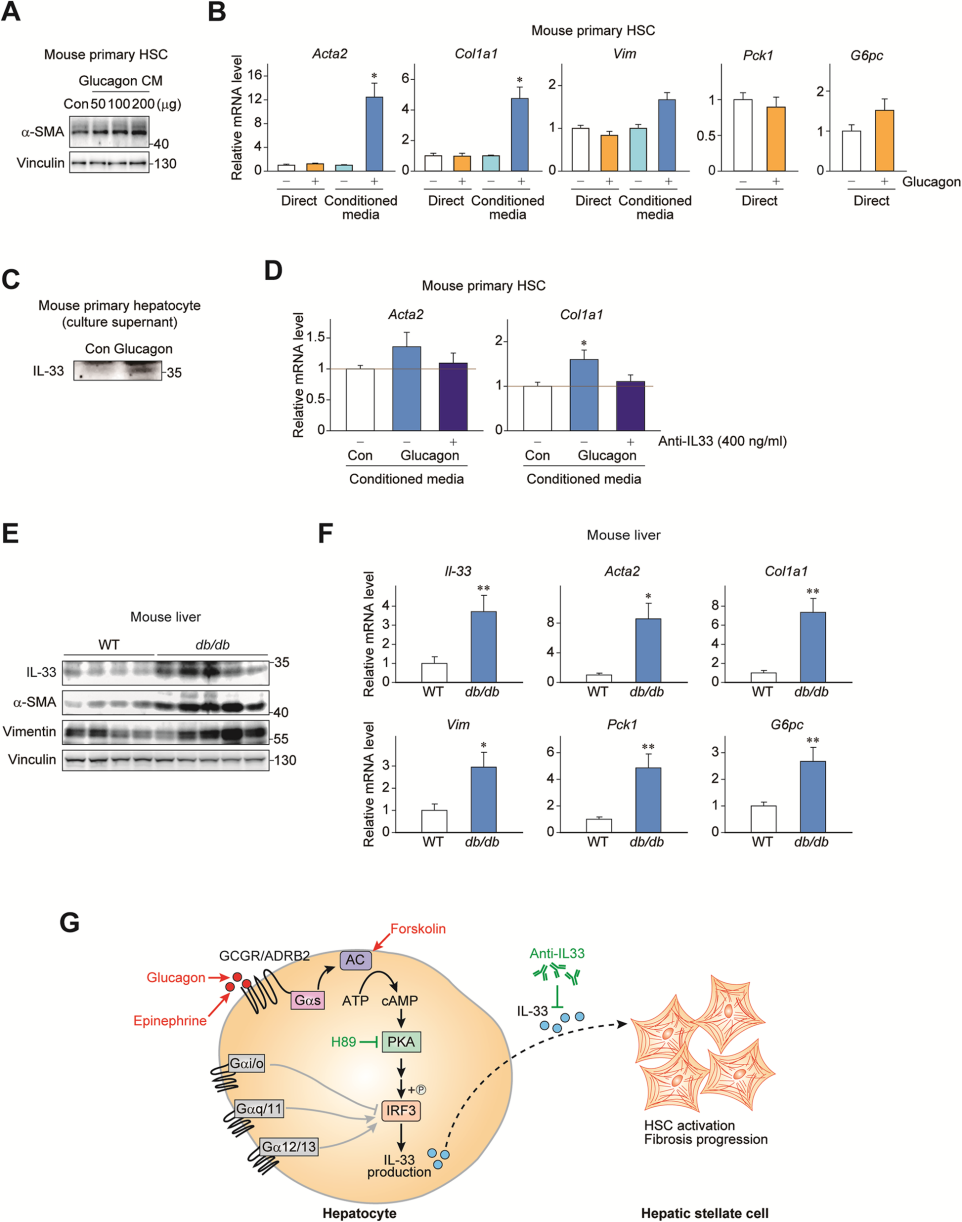
Gαs signaling in hepatocytes promotes HSC transdifferentiation through IL-33. **A** Mouse primary hepatocytes were treated with glucagon for 24 h. Secreted proteins were concentrated and applied to mouse primary hepatic stellate cells (day 1) in different doses for 24 h. **B** qRT-PCR analysis for HSC transactivation markers and gluconeogenic genes in primary HSCs with direct glucagon treatment or conditioned media from primary hepatocytes treated with 10 ng/ml glucagon (glucagon CM) or vehicle for 24 h (control CM). **C** Western blotting analysis of control CM and glucagon CM. **D** Mouse primary hepatocytes were treated with glucagon for 6 h. After washing with PBS, secreted proteins were collected for 24 h. qRT-PCR analysis was done from mouse primary HSCs (day 3) treated with respective conditioned media with or without IL-33 neutralizing antibody (400 ng/ml) for 24 h. **E** Western blot analysis of protein expression from livers of *db/db* or wild-type (WT) mice. **F** qRT-PCR analysis for hepatic gene expression (*n* = 4 for WT; *n* = 5 for *db/db* mice). **G** Overall scheme of this study. Statistical data were expressed as mean ± SEM; *p < 0.05 and **p < 0.01

Based on our molecular findings, we hypothesized that IL-33 induction by the Gαs-cAMP-PKA-IRF3 pathway may activate HSCs via intercellular signaling. Conditioned media from glucagon- or vehicle-treated mouse primary hepatocytes were collected and given to mouse primary HSCs. In line with our hypothesis, glucagon treatment in hepatocytes caused activation of HSCs presumably via soluble factor(s) in the conditioned media, as indicated by the increase of α-SMA protein expression (Fig. 6A). The finding was further corroborated with the expression of *Acta2* and *Col1a1* mRNA levels (Fig. 6B). In contrast, HSCs were irresponsive against direct glucagon treatment without hepatocytes, suggesting that the hepatocyte-to-HSC communication is crucial for glucagon effect on HSCs. Of note, IL-33 protein level was higher in the media of glucagon-treated hepatocytes, when compared to the those of non-treated control (Fig. 6C). To examine the role of IL-33, we interfered receptor binding of IL-33 using a neutralizing antibody. As a result, sequestration of IL-33 was able to block the hepatocyte to HSC communication and subsequent myofibroblastic transdifferentiation as evidenced by *Acta2* and *Col1a1* mRNA levels (Fig. 6D). Interestingly, elevated protein expression of hepatic IL-33, α-SMA and Vimentin was found in genetically obese *db/db* mice with constantly high serum glucagon levels [34], confirming the presence of signaling in a physiological setting (Fig. 6E). In support of the result, *db/db* mice also had increased mRNA levels of *IL33* along with the markers of HSC activation (*Acta2*, *Col1a1* and *Vim*) and glucagon signaling (*Pck1* and *G6pc*) (Fig. 6F). In summary, our results demonstrate that Gαs-cAMP-PKA pathway in hepatocytes activates IRF3, stimulates production of IL-33, and activates HSCs for liver fibrogenesis (Fig. 6G).

## Discussion

IRF3 is a protein that is involved in a wide range of cellular processes. Previous studies have primarily focused on how IRF3 is regulated downstream of cytosolic DNA sensing. Here, we newly demonstrate that GPCRs, which are involved in many cellular signaling pathways, can directly control IRF3 activity through Gα protein signaling. Our results have also revealed the specific mechanisms underlying GPCR-mediated IRF3 regulation. We discovered that IRF3 phosphorylation by Gαs signaling occurs independently of TBK1, a kinase known to be crucial for IRF3 activation in response to cytosolic DNA. Furthermore, the signaling stimulated production of IL-33 in hepatocytes and activated HSCs. Overall, our findings provide new insights into the regulatory mechanism of IRF3 and associated intercellular communication.

Our study introduces IRF3 as a downstream branch of GPCR signaling. GPCRs are essential regulators of a wide range of functions. All GPCRs converge on four different Gα proteins: Gαs, Gαi, Gαq/11, and Gα12/13. Initially, we focused on the role of Gαs because it showed robust IRF3 phosphorylation in an initial screen with constitutively active mutants and by ligands that activate Gαs-coupled receptors such as glucagon and adrenaline. In addition, our screen results suggest that Gα proteins other than Gαs may also impact IRF3. For example, Gαi inhibited IRF3 phosphorylation. The opposing effects of Gαs and Gαi are consistent with their opposing roles in downstream signaling. This supports our findings that cAMP plays a critical role in IRF3 regulation. Additionally, Gα11/15 and Gα12/13 also strongly phosphorylated IRF3, yet to a lesser extent. The broad regulation by different Gα proteins implies that a wide array of GPCR may have the potential to regulate IRF3. Since GPCRs have been the primary targets for recent development of therapeutic drugs [32], our results provide valuable pharmacological insights into the regulation of IRF3 and diseases involving activated IRF3 such as liver fibrosis.

Increased phosphorylation of hepatic IRF3 is observed in liver samples from fibrosis patients, and is associated with insulin resistance and abnormal blood glucose levels [9]. One suggested cause of this phosphorylation is the release of mitochondrial DNA into the cytoplasm, where cGAS recognizes mtDNA and generates cGAMP, activating IRF3 through STING-TBK1 [35]. However, although other tissues such as lung, rectum, pancreas, testis, prostate, bladder, heart and adipose tissue commonly show high expression, existence of STING in the liver is in debate [36], as the absence of STING expression in hepatocytes has been reported both in human and mice [37, 38]. This suggests that other upstream factor(s) are responsible for the regulation of IRF3 in hepatocytes. IRF3 has multiple phosphorylation sites that are controlled by different kinases including TBK1, JNK1, and MST1 [20–22]. However, most commercially available antibodies are only able to detect specific post-translational modifications that are previously identified, excluding novel modifications caused by unknown upstream factors. Phos-tag immunoblotting, used in this study, allowed us to discover the link between GPCR and IRF3 in an unbiased manner. In particular, when analyzed in a Phos-tag gel, phosphorylation was still observed in a mutant that is previously known to be “non-phosphorylatable”. On the other hand, phosphorylation did not occur when the SRD region was completely removed, leading to the identification of the SRD as the responsible site for Gαs-mediated regulation. Although the specific phosphorylation residue remains to be uncovered, a kinase for Tyr or His is expected as a link between PKA and IRF3, since mutation of all Ser and Thr sites in SRD could not fully block IRF3 phosphorylation. Thus, further investigations are warranted to identify the responsible kinase.

We have shown that conditioned media from glucagon-treated hepatocytes activates HSCs and that PKA signaling increases IL-33 expression in hepatocytes. However, it is counterintuitive in that normal liver never develops spontaneous fibrosis even in a prolonged fasting condition with high blood glucagon concentration [39]. Therefore, the newly found signaling is expected to take place only in pathological conditions but not during normal homeostasis. One possible explanation for this is that the potency of IL-33 is exaggerated during liver disease progression. IL-33 becomes more potent on its receptor activation when cleaved by neutrophil proteases, such as elastase and cathepsin G [40, 41]. Since neutrophils are actively infiltrated during the onset of liver fibrosis [42], secreted IL-33 may be more efficiently converted to its mature form. Indeed, the level of these proteases is known to be increased in livers with non-alcoholic steatohepatitis [43]. Many drug targets are not only present in diseased tissue but also perform vital functions in healthy tissues. In such cases, a drug that inhibits the target can potentially have side effects by interfering with the normal cellular function as well. Therefore, the aspect of IL-33 to exert greater potency in diseased liver suggests that targeting GPCR-IRF3-IL-33 signaling could be a safer therapeutic approach with a selectivity for diseased tissues.

## Conclusions

Our study reveals the potential for GPCRs to regulate IRF3 and the involvement of PKA-dependent phosphorylation in IRF3 regulation, which controls HSC transdifferentiation via IL-33. Interfering IRF3 activity by targeting GPCR will represent a novel pharmacological approach for liver fibrosis and other diseases associated with IRF3 dysregulation.

### Supplementary Information

**Additional file 1.**

## Data Availability

Not applicable.
